# Social Influence, Risk and Benefit Perceptions, and the Acceptability of Risky Energy Technologies: An Explanatory Model of Nuclear Power Versus Shale Gas

**DOI:** 10.1111/risa.13457

**Published:** 2020-02-13

**Authors:** Judith I. M. de Groot, Elisa Schweiger, Iljana Schubert

**Affiliations:** ^1^ Faculty of Economics and Business, Department of Marketing University of Groningen Groningen The Netherlands; ^2^ King's Business School London UK; ^3^ University of Basel Basel Switzerland

**Keywords:** Acceptability, energy technologies, risks perception, social influence, social networks

## Abstract

Risky energy technologies are often controversial and debates around them are polarized; in such debates public acceptability is key. Research on public acceptability has emphasized the importance of intrapersonal factors but has largely neglected the influence of interpersonal factors. In an online survey (*N* = 948) with a representative sample of the United Kingdom, we therefore integrate interpersonal factors (i.e., social influence as measured by social networks) with two risky energy technologies that differ in familiarity (nuclear power vs. shale gas) to examine how these factors explain risk and benefit perceptions and public acceptability. Findings show that benefit perceptions are key in explaining acceptability judgments. However, risk perceptions are more important when people are less familiar with the energy technology. Social network factors affect perceived risks and benefits associated with risky energy technology, hereby indirectly helping to form one's acceptability judgment toward the technology. This effect seems to be present regardless of the perceived familiarity with the energy technology. By integrating interpersonal with intrapersonal factors in an explanatory model, we show how the current “risk–benefit acceptability” model used in risk research can be further developed to advance the current understanding of acceptability formation.

## 1 INTRODUCTION

The U.K. energy market is facing an energy trilemma of secure energy supply, affordable energy, and sustainable technologies (World Energy Council, 2018). With the increase of oil prices, reduced fossil fuel reserves, the need of independent energy extraction, and climate change, the U.K. government has reassessed the need to invest in nuclear power (NP) and alternative methods of securing energy supply with limited CO_2_ emissions, such as shale gas (SG) extraction (BBC News, 2013, 2014).

NP and SG have caused great controversy worldwide, and specifically in the United Kingdom (Department for Business, Energy and Industrial Strategy, 2017). Despite growing public acceptability for NP stations over the last few decades, public acceptability of NP in the United Kingdom and other European countries remains ambivalent. Proponents view NP as a very clean energy source with few greenhouse gas emissions (International Atomic Agency, 2018), while opponents point to the problem of nuclear waste disposal and the fear of power plant accidents (Li, Fuhrmann, Early, & Vedlitz, 2012). Data from nationally representative British surveys over the last years have shown that NP retains one of the lowest acceptance rates among different sources of energy generation (Department for Business, Energy and Industrial Strategy, 2017).

More recently, SG (or “fracking”) has caused great controversy in the United Kingdom (Department for Business, Energy and Industrial Strategy, 2018; The Guardian, 2018). Proponents point to the United States where the technique is common and has strengthened their economy while securing an independent energy supply (Stevens, 2012). They also emphasize the economic advantage of obtaining cheap energy (O'Hara, Humphrey, Andersson‐Hudson, & Knight, 2016). Opponents stress the negative environmental impact, including the increased likelihood of earthquakes, the contamination and depletion of ground and fresh water due to chemicals used in the fracking fluid, and the impact on air quality (O'Hara et al., 2016; Williams, Macnaghten, Davies, & Curtis, 2017). A national U.K. survey on public opinion in relation to SG showed that of the people who felt knowledgeable enough to form an opinion about SG, 33% opposed and 16% supported fracking (Department for Business, Energy and Industrial Strategy, 2017).

The energy trilemma requires urgent decisions by policy makers. Such decisions are highly influenced by public acceptability as opposition toward energy technologies has repeatedly shown to impact political decisions. For example, rejection of NP plants peaked after the Fukushima accident (Visschers & Siegrist, 2013), causing German national policy responses to favor renewable energy technologies in the lead up to regional elections (Wittneben, 2012). Similarly, fracking was delayed until 2018 in the United Kingdom because it was linked to earthquakes in 2011; and the first fracking site in Lancashire has faced large protests since the beginning (BBC News, 2018). Hence, understanding public acceptability is vital in establishing energy security policies, as they drive decisions regarding the future of the U.K. energy mix (Poortinga, Aoyagi, & Pidgeon, 2013).

Public acceptability of risky technologies can be regarded as an attitude (De Groot, Steg, & Poortinga, 2013). Attitudes are psychological tendencies to evaluate an attitude object (i.e., energy technology) through weighting the costs (or “risks’) and benefits of a specific object or behavior (Ajzen, 1985). The higher the perceived risks and the lower the perceived benefits of an energy technology, the less likely people are to evaluate that specific technology positively, and vice versa (Siegrist & Cvetkovich, 2000; Siegrist & Sütterlin, 2014). The affect heuristic provides an explanation for the strong intercorrelation between risks and benefits: people base their risk assessment on an initial overall evaluation (“affect”) and adjust their specific beliefs about the risks and benefits to fit into their preconceived view (Finucane, Alhakami, Slovic, & Johnson, 2000). Even though risk and benefit perceptions are strongly correlated, most research includes both risk and benefit perceptions in relation to the acceptability of risky attitude objects (Bearth & Siegrist, 2016; Bearth, Cousin, & Siegrist, 2014; Bearth, Miesler, & Siegrist, 2017; Dreyer, Polis, & Jenkins, 2017; Ho & Watanabe, 2018; Hubert, Blut, Brock, Backhaus, & Eberhardt, 2017; Poortvliet, Sanders, Weijma, & De Vries, 2018; Siegrist, Stampfli, Kastenholz, & Keller, 2008), including energy technologies (Ho et al., 2018; Lienert, Sütterlin, & Siegrist, 2015; Visschers, Keller, & Siegrist, 2011; Whitfield, Rosa, Dan, & Dietz, 2009).

Research on risk and benefit perceptions and public acceptability of risky energy technologies has extensively focused on the cognitive and attitudinal processes at an intrapersonal level (De Groot & Steg, 2010; Slimak & Dietz, 2006; Slovic, Fischhoff, & Lichenstein, 1982), such as, values (De Groot et al., 2013; Whitfield et al., 2009), and trust and uncertainty (Knoblauch, Stauffacher, & Trutnevyte, 2018; Siegrist & Cvetkovich, 2000; Terwel, Harinck, Ellemers, & Daamen, 2011). However, far less attention has been given to the impact of interpersonal influences, including social influence (Bickerstaff, 2004; Helgeson, van der Linden, & Chabay, 2012; Howell et al., 2017). This is surprising seeing as social influence is known to reduce conflict and uncertainty within the individual through the development of shared attitudes (Friedkin, 2001).

The present study examines an explanatory model of the acceptability of NP and SG in a U.K. context by integrating social influence in the existing “risk–benefit acceptability” model. Furthermore, social influence might impact risk and benefit perceptions and public acceptability differently, depending on how familiar individuals are with an energy technology. Therefore, we compare the slightly more familiar risky energy technology of NP to SG, which people seem to be somewhat less familiar with (Department for Business, Energy and Industrial Strategy, 2017, 2018). Examining how intra‐ and interpersonal processes explain public acceptability will help to further develop the “risk–benefit acceptability’ model used in the field.

### 1.1 Risk and Benefit Perceptions and Public Acceptability

Previous research shows that both risk and benefit perceptions are relevant in explaining the acceptability of risky energy technologies (Dreyer et al., 2017; Howell et al., 2017; Visschers et al., 2011). This assumption has especially been validated in the field of NP (De Groot et al., 2013; Greenberg & Truelove, 2011; Keller, Visschers, & Siegrist, 2012; see Ho et al., 2018 for an overview). For example, a recent meta‐analysis including 34 studies examining public perceptions toward NP showed that both benefit and, although to a lesser extent, risk perceptions were important predictors for the acceptability of NP (Ho et al., 2018). Research investigating the processes of how risk and benefit perceptions influence acceptability of SG is slowly growing as well (Christenson, Goldfarb, & Douglas, 2017; Howell et al., 2017; O'Connor & Fredericks, 2018; Pollard & Rose, 2018; Thomas, Partridge, Harthorn, & Pidgeon, 2017). For example, a multilevel analysis including both intrapersonal‐ and state‐level factors found both risk and benefit perceptions are important intrapersonal factors influencing the acceptability of fracking in the United States (Howell et al., 2017). The few studies that have focused on the processes underlying risk and benefit perceptions and the acceptability of SG imply that both risk and benefit perceptions are important when explaining the acceptability of the less familiar energy technology of SG. Like with NP, benefit perceptions seem to be a stronger predictor for the acceptability of SG, although with SG the advantages seem to be easily forgotten when people are confronted with the risks as well (Thomas et al., 2017). However, the sparse amount of studies testing these relationships makes conclusions tentative only.

This study will further validate the “risk–benefit acceptability” model as proposed in risk research. We put forward the following hypotheses:

**Hypothesis 1**: Risk and benefit perceptions toward risky energy technologies (NP and SG) will explain the public acceptability of the technology.

That is, higher risk perceptions decrease the acceptability of the respective energy technologies (Hypothesis 1a); lower benefit perceptions decrease the acceptability of the respective energy technologies (Hypothesis 1b).

**Hypothesis 2**: Benefit perceptions will relate more strongly to the acceptability of risky energy technologies than risk perceptions.

### 1.2 Social Influence, Risk Perception, and Public Acceptability

Interpersonal influences are known to reduce conflict and uncertainty within the individual through the development of shared attitudes (Friedkin, 2001). Interpersonal relationships link social actors that share beliefs and influence one another in attitude formation (Helgeson et al., 2012). In the late 1980s, scholars already acknowledged that social influences among friends, family members, or coworkers, influence the process of shaping attitudes toward risky issues (Kasperson, Renn, Slovic, Brown, & Emel, 1988; Slovic, 1987). Given the remarkably large scope of social phenomena that are shaped by social influence (Latane, 1981), it is surprising that interpersonal determinants, such as social influence, have been less focused on in risk research. Moreover, research that has included a social dimension, has done so from an intrapersonal perspective only, in the form of social norms (e.g., Featherman & Hajli, 2016; Hilverda & Kuttschreuter, 2018; Silva, Jenkins‐Smith, & Barke, 2007; Trumbo, 2018). As social norms have been conceptualized as “personal beliefs” in relation to what is commonly accepted or commonly done in a specific social context (Cialdini, Reno, & Kallgren, 1990), it therefore still treats social influence as a typical intrapersonal rather than interpersonal factor.

Investigating social networks has been one way to examine the effects of social influence on attitude formation from an interpersonal perspective. Social networks are dyadic ties (relationships) between actors (individuals or organizations) that are characterized by resource exchange (Haythornthwaite, 1996). These resources may include social support, information exchange, or influence. Social network theory conceptualizes actors and social structures as relational in nature and investigates the outcomes of inter‐ and intragroup processes (Borgatti & Halgin, 2011). Social networks occur in many different settings, such as different stakeholder groups (Brooks, Hogan, Ellison, Lampe, & Vitak, 2014), with people belonging to a variety of different networks at the time. Within these networks, individuals are interconnected to different degrees and the number of network ties varies.

Research on social networks shows that merely talking about a risky attitude object, such as energy technologies, with others in your network, and increasing your knowledge about this attitude topic and other's belief system, can play an important role in influencing your own beliefs (i.e., risk and benefit perceptions) and attitudes (i.e., acceptability) (cf. Scott, 2017). However, only few studies have examined the relationship between social network characteristics and risk and benefit perceptions (Kohler, Behrman, & Watkins, 2007; Muter, Gore, & Riley, 2013; Scherer & Cho, 2003). Scherer and Cho (2003) examined a social network contagion theory of risk perception to account more adequately for social or social–structural variables in environmental conflicts, such as hazardous waste side cleanups. Their findings showed that people who are in frequent contact with one another (in their network) are also more likely to share similar attitudes and beliefs regarding an environmental conflict over a hazardous waste site cleanup. The social network contagion theory of risk perception was further supported by Kohler et al. (2007) who showed, in a longitudinal study, that the risk perception in one's social network in relation to catching AIDS influences the extent to which someone believes that they are at risk themselves.

Although these studies seem to suggest that social influence, as measured with social network characteristics, likely relates to beliefs and attitudes of risky attitude objects, they need to be expanded in three ways to further develop the field of social influence and risk research. First, acknowledging that social influences partially constitute the process behind shaping attitudes toward risky issues (Kasperson et al., 1988; Slovic, 1987) raises the question: To what extent and under what conditions does social influence impact the acceptability of highly controversial and debated energy technologies, such as NP and SG? Previous studies incorporating social influence to examine risk perception and evaluation have focused on risk issues that are more observable and identifiable, such as health risks (Kohler et al., 2007). In contrast to most health‐related risks, risks associated with energy technologies, especially the risks that are associated with climate change, cannot be easily observed and identified (Helgeson et al., 2012), which makes it difficult for lay people to estimate the risks associated with it (Kasperson & Ram, 2013). This study focuses on energy technologies that have been typically associated with climate change (Poortinga et al., 2013), hereby extending our knowledge on the extent to which social influence is relevant in a different context.

Second, the few studies focusing on social influence in relation to risk perception have largely neglected the previously well‐established “risk–benefit acceptability” model in risk research (Ho et al., 2018; Lienert et al., 2015; Siegrist & Sütterlin, 2014; Visschers et al., 2011). That is, they have focused on risk perception rather than on how risk perception influences evaluations (i.e., acceptability). Consequently, we are still left in the dark on how social influence fits into the model.

Third, the studies on social influence in relation to risk perceptions have only focused on general social network ties within one's network. However, not only the presence of social influence but also the number of close network partners and their perception of risks and benefits can influence people (Haythornthwaite, 1996). For example, a person who has a lot of close network partners supporting NP or SG, may be more likely to evaluate that type of energy technology positively and deem it as less risky than someone who has close network partners that oppose or have no opinion about NP or SG. Similarly, if you have never talked to your close friends about NP or SG, they may have less influence on how you form your opinion of the energy technologies. However, if you speak to your close friends about risky energy technologies, they can influence your risk and benefit perceptions toward these technologies in different ways, depending on how they talk about it (i.e., emphasizing all the risks or all the benefits). This study integrates these two social network characteristics to provide a more comprehensive view of how social influence affects risk and benefit perceptions and acceptability.

This research determines the extent to which social influence is important in explaining one's risk and benefit perceptions toward the high‐risk energy technologies of NP and SG. By incorporating social network analysis (SNA) to measure social influence, this study will be the first to provide empirical insights about the extent to which social influence is relevant for explaining the acceptability of risky technologies. Based on previous research (Kohler et al., 2007; Muter et al., 2013; Scherer & Cho, 2003), we assume:

**Hypothesis 3**: One's social network affects risk and benefit perceptions of risky energy technologies (NP and SG).

That is, the perceived risks toward a risky energy technology will be higher the less the individual perceives peer support from their social network for the technology (Hypothesis 3a). Furthermore, the perceived risks toward a risky technology will depend on the extent to which the individual talks with their peers about the technologies (Hypothesis 3b). The perceived benefits toward a risky energy technology will be higher the more the individual perceives peer support from their social network for the technology (Hypothesis 3c). Finally, the perceived benefits toward a risky technology depend on the extent to which the individual talks with their peers about the technologies (Hypothesis 3d).

More specifically, as previous research suggests that most determinants of risky energy technologies’ acceptability exert their influence via risk and benefit perceptions (Siegrist & Cvetkovich, 2000), we hypothesize a mediation effect:

**Hypothesis 4**: Social influence affects the acceptability of the risky energy technology (NP and SG) mainly indirectly, via risk (Hypothesis 4a) and benefit (Hypothesis 4b) perceptions.

### 1.3 The Impact of the Familiarity of NP and SG on Risk Perception and Acceptability

People may differ slightly in how familiar they are with NP and SG. Being less familiar with a risky technology results in an increased uncertainty associated with them (Department for Business, Energy and Industrial Strategy, 2017, 2018; O'Hara et al., 2016). Although NP and its associated risks and benefits have been known for decades (Ho et al., 2018), SG has only more recently attracted public attention (Department for Business, Energy and Industrial Strategy, 2017). Although the awareness toward SG has grown over the last years (Department for Business, Energy and Industrial Strategy, 2018), most people still perceive themselves to be slightly less knowledgeable about the topic than about NP (Department for Business, Energy and Industrial Strategy, 2017; Williams et al., 2017).

Differences in the perceived familiarity between NP and SG may also result in dissimilarities in the importance of risk and benefit perceptions on the acceptability of these risky energy technologies. Heuristics, such as the negativity bias (Ahluwalia, 2002; Baumeister, Bratslavsky, Finkenauer, & Vohs, 2001), suggest that individuals tend to weigh information regarding the presence of risks more strongly than when presented with neutral or positive information. Hence, even though in an absolute sense benefit perceptions are more strongly related to the acceptability of risky technologies than risk perceptions (Ho et al., 2018), the negativity bias suggests that risk perceptions are easier “to make salient” when people are confronted with information. Indeed, research into risk perceptions shows that emphasizing risks, rather than benefits or neutral information associated with *unfamiliar* risky attitude objects, such as nanotechnology (Cobb, 2005), vaccination risks (Betsch, Haase, Renkewitz, & Schmid, 2015), or general health dangers (Siegrist & Cvetkovich, 2001), influences evaluations of them more strongly. Van Giesen, Fischer, and Van Trijp (2018) provide a possible explanation of why the negativity bias works differently depending on the familiarity of an attitude object. In a longitudinal study toward an emerging risky technology (nanotechnology), they found that acceptability levels were less reliant on the affective than the cognitive components of attitude formation over time. This effect occurred because fewer knowledge structures were in place to rationalize negative information (Van Giesen et al., 2018). Over time and with the increase of knowledge, more structures were available causing attitudes to be formed as a combination of affect and cognitions. Based on this, we hypothesize:

**Hypothesis 5**: Risk and benefit perceptions will relate differently to the acceptability of NP as compared to SG.

That is, risk perceptions will affect the acceptability of the more familiar technology of NP less strongly than of the less familiar technology of SG (Hypothesis 5a); benefit perceptions will affect the acceptability of the more familiar technology of NP more strongly than of the less familiar technology of SG (Hypothesis 5b).

### 1.4 Exploring the Effect of Social Influence on Risk and Benefit Perceptions for Technologies Differing in Familiarity

In addition to our argument that social influence factors are important to understand risk and benefits perceptions and acceptability of risky energy technologies, we also argue that differences in familiarity between NP and SG impact the nature of these relationships. Social interactions are the key mechanism through which individuals validate their attitudes under conditions of uncertainty and conflict (Moussaïd, Kämmer, Analytis, & Neth, 2013). Although both NP and SG are risky technologies associated with ambivalence, the difference in familiarity may result in more perceived uncertainty with the less familiar technology of SG.

As argued above, an important distinction between NP and SG is the extent to which people are familiar with these energy technologies. Social influence, as conceptualized through social network characteristics, could impact risk and acceptability judgments of technologies that differ in how familiar people are with them. For example, Christenson et al. (2017) have shown how malleable the acceptability of SG is when citizens are less familiar with the technology. These results imply that beliefs about risks and benefits, and consequently acceptability‐judgments, are more easily influenced by the opinion of close network members. Therefore, it could be argued that people's risk and benefit beliefs in relation to SG (and their acceptability judgment) will be more strongly influenced by the attitude of their close peers than their perceptions in relation to NP as they are likely more familiar with this technology.

This research includes an explorative research question as a first step to understand whether social networks differently influence: (1) a person's risk perceptions directly; and (2) acceptability indirectly, depending on the energy technology (NP and SG; *RQ1*):

How do social network factors affect risk and benefit perceptions, and acceptability differently for risky technologies that differ in familiarity (NP vs. SG)?

### 1.5 The Present Study

**Figure 1:**
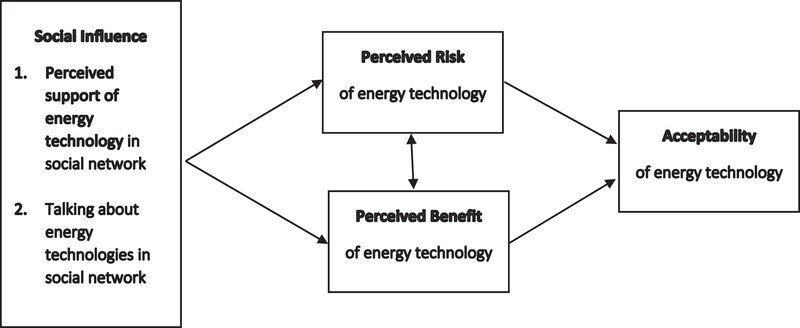
Model of the acceptability of risky energy technologies.

To test the presented hypotheses and *RQ1*, we will present an explanatory model to investigate the importance of social influence on the formation of risk and benefit perceptions directly and indirectly the acceptability of NP and SG. The baseline model for both energy technologies is presented in Fig. 1. As social influence might be differently related to risk and benefit perceptions and public acceptability, depending on how familiar individuals are with an energy technology, we compare the more familiar energy technology of NP with the less familiar technology of SG.

## 2 METHODS

### 2.1 Sampling and Participants

A survey company distributed an online questionnaire to selected panelists from the British population of over 18 years of age. We collected 1,000 responses of which 52 participants were removed because they failed to follow the instructions of the quality‐fail question. The quality‐fail question asked participants to choose a specific answer. If they failed to do this it indicated that they did not read the survey questions properly. Therefore, a total of 948 participants were included in the remaining analysis. The sample's mean age was 49.90 years old (*SD* = 14.08), and 52% were female. Distributions of age and gender (UK Statistics, 2011), education (Census, 2011), and income (Belfield, Cribb, Hood, & Joyce, 2014) were compared with data of the U.K. population. Comparisons of our data on these sociodemographic variables indicated that our sample of panelists reflected a reasonable representation of the adult U.K. population.

### 2.2 Questionnaire and Measures

An online questionnaire was designed using a survey programming tool. Respondents answered questions regarding their familiarity with NP and SG, followed by measuring risk and benefit perceptions of NP and SG, and an acceptability judgment of these two energy technologies. Next, participants were asked to provide information of their social network and their social network's perceptions of NP and SG. The measures of the main variables are described in more detail (Table I).1The reviewers voiced concerns about the measure of the dependent variable “acceptability” and the independent variable “familiarity.” To address these concerns, we decided to collect some more data. Our additional data collection with amended items showed no obvious differences from our main analyses as reported in this article. Hence, our operationalizations of constructs have not influenced the main conclusions of our reported findings. For a summary of this additional study, please see the Supporting Information.


**Table I:** Constructs of the Questionnaire, Respective Items, and the Sources They Were Adapted From

Intrapersonal Variables		Source
Acceptability	The United Kingdom needs a lot of electricity; people should therefore accept nuclear power.	Visschers et al. (2011)
	*We can give up nuclear power without any problem*.	Visschers et al. (2011)
	I reluctantly accept that we will need nuclear power to help combat climate change.	Corber et al. (2011)
	I am in favor of nuclear power to be part of the of the United Kingdom's energy mix in 2025.	O'Hara et al. (2014)
	I reluctantly accept that we will need nuclear power to help improve energy security in the United Kingdom.	Corber et al. (2011)
Risk perception	The risk of accidents in the U.K. nuclear power industry is minimal.	Visschers et al. (2011)
	U.K. nuclear power stations are safe.	Visschers et al. (2011)
	Nuclear power degrades animals, plants, land, and water.	Greenberg (2009)
	In general, how risky do you consider the use of nuclear power to be to the society as a whole?*	Finucane, Alhakami, Slovic, & Johnson (2000)
Benefit perception	Nuclear power has a positive impact on climate mitigation.	Visschers et al. (2011)
	Nuclear power provides secure energy supply.	Visschers et al. (2011)
	Nuclear power results in cheap energy.	O'Hara et al. (2014)
	In general, how beneficial do you consider the use of nuclear power to be to society as a whole?*	Finucane, Alhakami, Slovic, & Johnson (2000)
Interpersonal Variables		
Name generator:		
Affect approach	Who belongs to your closest circle of people you interact with and spend a lot of time with? These may include people from your family, circle of friends, or people from your professional life (i.e., university, school, work, sport clubs) with whom you discuss personal matters and have spent a substantial amount of time with within the past six months.	Marsden (2005)
Exchange approach	With whom, out of the people that you have already listed, have you talked about nuclear power or shale gas/fracking? You can click on multiple people.	
Name interpreter:	Is the following person a proponent of nuclear power?	Carrington et al. (2005)
	How close are you to each of the above‐mentioned people?	
	How long have you known these people in years?	
	How risky does the following person consider the use of nuclear power to be to the society as a whole?	
	Who influences your perspective of nuclear power? Please check all that apply.	
Familiarity	How familiar are you with the risks and benefits of nuclear power?	Boudet et al. (2014)
	How much have you ever heard or read about nuclear power?	Boudet et al. (2014)

*Note*: The items are shown for nuclear power. The questions assessing shale gas used the same wording only replacing “nuclear power” with “shale gas.” Intrapersonal variables were all measured on a Likert scale ranging from 1 “strongly agree” to 5 “strongly disagree.” The item in *italics* has been deleted in the final measurement model because of low cross‐loadings (<0.05) with the other construct items. Items including an asterisk symbol were all measured on a different Likert scale, that is, risk items were measured on a scale ranging from 1 “not at all risky” to 5 “extremely risky”; benefit items were measured on a scale ranging from 1 “not at all beneficial” to 5 “extremely beneficial.”

#### 2.2.1 Acceptability

Five items for each energy technology measured the acceptability of NP and SG in the United Kingdom. All items were measured on a five‐point Likert scale ranging from 1 “strongly agree” to 5 “strongly disagree.” Initial correlations between the constructs and items showed that one item of acceptability (“We can give up NP without any problem.”) showed low cross correlations with the other acceptability items and with the construct for both NP and SG. Therefore, this item was deleted prior to the final evaluation of the measurement models. The use of SG (*M* = 3.00, *SD* = 0.80, Cronbach's *α* = 0.89) was evaluated as somewhat less acceptable than NP (*M* = 3.36, *SD* = 0.79, Cronbach's *α* = 0.86).

#### 2.2.2 Risk and Benefit Perceptions

Four items measured participants’ perceived risks for NP and for SG, including beliefs in relation to accident risks, safety concerns, environmental degradation, and general risk to society. Four items measured participants’ beliefs related to the perceived benefits of NP and SG, such as climate change mitigation, secure energy supply, affordable energy, and their general benefit to society. Risk and benefit items were measured on a five‐point Likert scale ranging from 1 “strongly agree” to 5 “strongly disagree” (with and exception of the two general items: “How risky do you consider the use of NP to be to society as a whole?” and “How beneficial do you consider the use of NP to be to society as a whole?”; see Table I). The means and standard deviations for risk and benefit perceptions were relatively similar for both SG and NP. The means indicated that participants generally neither agreed, nor disagreed that SG/NP was risky (SG risks *M* = 3.17, *SD* = 0.82, Cronbach's *α* = 0.83; NP risks *M* = 2.85, *SD* = 0.81, Cronbach's *α* = 0.82), or beneficial (SG benefits *M* = 2.95, *SD* = 0.83, Cronbach's *α* = 0.85; NP benefits *M* = 3.24; *SD* = 0.80, Cronbach's *α* = 0.81).

#### 2.2.3 Social Influence

One of the ways in which social influence has been examined in the past is through SNA. SNA helps to analyze the content, patterns, and dynamics within social groups by statistically analyzing connections between different interdependent actors within a group (Wasserman & Faust, 1994). There are two main types of SNA. One of them investigates whole or complete social networks by mapping the interconnections between all social network actors of a specific network group to understand group dynamics, how social capital is achieved (Nahapiet & Sumantra, 1998), and, how weak ties are being used (Granovetter, 1973). The other type of SNA is the so‐called egocentric analysis, which focuses on the social network of an individual (ego) and how his or her social network affects an individual also referred to as *the ego*. An ego's social network may include peer groups such as friends, family, and colleagues, who are called *alters*. Thus, in SNA, “the individual” is referred to as “the ego” and “the individual's peers” are referred to as “their alters.”

During an ego SNA, beliefs and attitudes of alters can be assessed through questioning the individual about their perceived attitudes and behavior. Such an analysis is more appropriate and relevant for the proposed study than a complete SNA, because an individual needs to perceive his/her alters’ attitudes in order to be influenced by them (Ajzen, 1985). Consequently, the perceived alters’ acceptability toward NP and SG is superior to the alters’ actual evaluation of it (Visser & Mirabile, 2004). Egocentric analysis enables the collection of a larger sample of different ego networks in comparison to complete SNA, thus resulting in a more coherent analysis of how alters influence an individual's perceptions. Therefore, we applied an ego‐network analysis by asking participants to rate the attitudes of their network peers toward energy technologies.

Participants’ social network alters and their characteristics were retrieved in two stages: (1) generating names, followed by (2) interpretation questions. This two‐step approach enabled us to assess a person's perception of the attitudes prevalent in his or her social network (Table I).

*Name generator questions* were used to obtain a participant's list of social network peers (i.e., alters). Multiple name generator questions were employed in this study to increase the reliability of the social network data (Marin & Wellman, 2011). The first name generator question we used was based on the affect approach, which meant we asked about alters that were high in affective value to the participant (Marsden, 2005). This approach enabled us to collect alters from a wide variety of social groups such as family, friends, or colleagues. To further narrow down one's social network to alters with whom participants exchanged some kind of information over the topic of NP or SG, we used the exchange approach (Carrington, Scott, & Wasserman, 2005; Marsden, 2005). Both name generator approaches allowed for the collection of information regarding social network's partners that (1) were close to the ego, or (2) communicated with the ego about NP or SG.

In the second step of collecting social network data, so called “name interpreter questions” were used. These questions gathered additional information from the network alters and the alters’ relationship with the participant (Marin & Hampton, 2007). We regarded the following two specific social influence factors as important for the aim of the present study:

(1) The number of people you have talked to about NP and SG in one's social network. Via the name generator (Marsden, 2005), participants were asked to either impart first names or initials of the alters to make them feel more confident about sharing personal data. Participants could name between two and 15 social network partners. On average, participants named six social network members (*M* = 6.07, *SD* = 3.06). After participants indicated, via the name generator, their closest circle of people, they were asked to state with whom they have had a discussion about NP or SG. On average, participants talked to 1.60 people about NP or SG (*SD* = 1.97).

(2) Social Network Index (SNI). SNI measured the extent to which the individual (the ‘ego’) perceived support for NP/SG in one's social network. Participants were asked to indicate for each person of their social network whether that person was a proponent (Pro) or opponent (Con) of NP or SG. They could also indicate if they did not know (DNK) the person's attitude toward the energy technologies of NP and SG. Based on this data, we created an index for the overall support for NP and SG participants’ perceived in their social network. The index ranged from 0 to 1. A zero indicated that all members of the participant's network opposed the energy technology, while a one indicated that everyone in the network supported the technology. A value of 0.50 indicated that the number of positive and negative social influence opinions equaled each other out or that people in the network did not express their attitude toward the energy technologies. The index was calculated using the following formula: ((Con × 0) + (DNK × 0.5) + (Pro × 1)): (Con + DNK + Pro). Thus, we calculated a weighted average of the opinions prevalent in a participant's immediate social network. The mean of the perceived opinions within one's social network were almost neutral for NP (*M* = 0.49, *SD* = 0.23) and neutral to slightly opposing for SG (*M* = 0.44, *SD* = 0.23).

#### 2.2.4 Familiarity

Familiarity with the energy technologies was measured to check our assumption that people are in general more familiar with NP than with SG. Participants rated their subjective perception of knowledge and the amount they had heard or read about NP and SG on a Likert scale ranging from 1 “not at all familiar” to 5 “extremely familiar” (Boudet et al., 2014). As expected, participants indicated they were slightly more familiar with NP (*M* = 2.86, *SD* = 1.00, *α* = 0.88) than with SG (*M* = 2.53, *SD* = 1.03, *α* = 0.89. The difference was significant, with a medium to strong effect size (*t*(947) = 12.99, *p* < 0.001; Cohen's *d* = 0.33, 95% confidence intervals [CIs]: 0.45–0.20). For the remaining analyses, we will therefore report and compare both models of NP and SG separately. This can show us whether social influence explains risk and benefit perceptions and acceptability differently depending on the familiarity of the energy technology.

### 2.3 Analyses

Partial least squares‐structural equation modeling (PLS‐SEM) was used to estimate both models using StataSE15. PLS‐SEM is a composite‐based approach to SEM that combines principal component analysis and regression to explain the variance of the target constructs in a structural model (Chin, 2010). All path coefficients and specific item loadings are simultaneously measured in the context of the specified model. As regression analysis inflates measurement errors, PLS‐SEM is an effective tool to test the proposed relationships among the constructs by reducing Type II errors (Hair, Hult, Ringle, & Sarstedt, 2017). Furthermore, our study included both reflective (i.e., risk perceptions; Mode A), formative (i.e., benefit perceptions, acceptability; Mode B), and single‐item constructs (i.e., social influence factors). PLS‐SEM seemed more appropriate than covariance‐based SEM, as it allows researchers to eliminate biases and inconsistent parameter estimates when dealing with these more complex models (Hair et al., 2017).

We conducted a two‐step procedure. The first step included evaluating the measurement models of NP and SG. Indicator reliability was assessed with item loadings of the four risk perception items, all loadings were deemed acceptable (>0.50). We assessed construct reliability with Cronbach's alpha and Dillon‐Goldstein's rho. Risk perception showed a high internal consistency in both models, with Cronbach alpha's (*α*_NP_ = 0.82; *α*_SG_ = 0.84) and Diller‐Goldstein ƿ values (ƿ_NP_ = 0.88; ƿ_SG_ = 0.89) above the acceptable 0.70 criterion (Hair et al., 2017). Convergent validity was tested with the average variance extracted (AVE) and by checking the standardized loadings of the construct. The AVE for risk perception was well above the recommended 0.50 for both models (0.64 for NP and 0.67 for SG) (Chin, 2010). Standardized loadings all exceeded the recommended 0.70 (Chin, 2010). Finally, discriminant validity was confirmed for the formative constructs, with VIF scores lower than 4 (Hair et al., 2017). Discriminant validity was checked with the Fornell‐Larcker Criterion (i.e., comparing the square root of the AVE of the construct risk perception to its correlations with other constructs (Fornell & Larcker, 1981) for the reflective construct “risk perception.” The AVE of risk perception was higher than the correlations with all other constructs for both models, except for one. The AVE of the construct risk perception for SG was 0.67 while the correlation between risk perception and acceptability was 0.68, indicating a potential issue with discriminant validity. We decided that the discriminant validity was acceptable for the purpose of the present study, because (1) the difference between the AVE and correlation was negligible, with less than 0.01 difference; and, (2) theoretically, scholars have measured risk, benefit and acceptability in a similar way and regarded them as distinct concepts (e.g., Finucane et al., 2000; Visschers et al., 2011). As there were no serious issues related to reliability and validity of the measurement models of NP and SG, we will only report the structural model in our results (Step 2).

Step 2 showed the structural models for NP and SG, which enabled us to test our hypotheses and *RQ1*. Convergence was achieved after 10 iterations for both models. PLS‐SEM does not assume a specific data distribution; therefore, no formal fit indices are used. We used bootstrapping with 200 replications as a resampling technique to derive the parameters’ standard errors (Ali, Rasoolimanesh, Sarstedt, Ringle, & Ryu, 2018). We reported the *R* ^2^ of the endogenous constructs and included the effect sizes (*ƒ* ^2^) for the *R* ^2^. Threshold values of 0.02, 0.15, and 0.35 indicate weak, moderate and strong effects respectively (Cohen, 1988). We also reported the standardized path coefficient estimates including significance levels, and 95% CI where appropriate. We considered the path coefficients models to be significantly different when the CIs of these weights overlapped no more than half of the distance of one side on a CI (Masson & Loftus, 2003). We conducted a mediation analysis to test mediation effects and a multigroup analysis to compare the model of NP with SG (Venturini & Mehmetoglu, 2019).

## 3 RESULTS

### 3.1 Social Influence, Risk and Benefit Perceptions, and Acceptability of Energy Technologies

**Figure 2:**
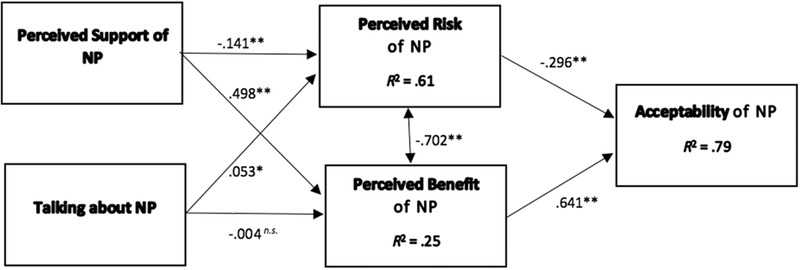
Estimated model with standardized regression weights for nuclear power (NP), *N* = 948. *p < 0.01; **p < 0.001; n.s. = not significant.

**Figure 3:**
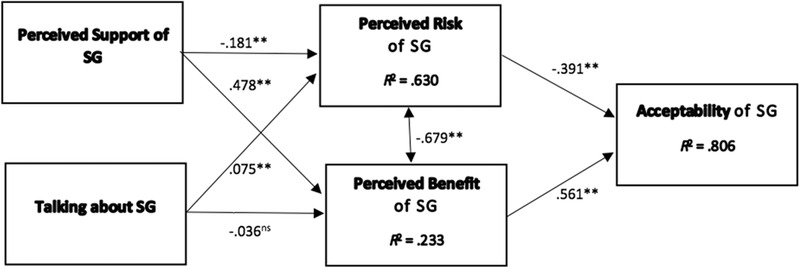
Estimated model with standardized regression weights for shale gas (SG), *N* = 948. *p < 0.01; **p < 0.001; n.s. = not significant.

Figs. 2 and 3 show the results of the structural model estimation and evaluation for the relationships between the two social influence factors, risk and benefit perceptions and acceptability for NP and SG. Both of the proposed models’ showed a strong effect in predicting acceptability (NP: *R* ^2^ = 0.79, *p* < 0.001; *ƒ* ^2^ = 3.76; SG: *R* ^2^ = 0.81, *p* < 0 001; *ƒ* ^2^ = 4.26).

In line with Hypothesis 1, the less risks (*β*_NP_ = –0.30, *p* < 0.001; *β*_SG_ = –0.39, *p* < 0.001; Hypothesis 1a), and the more benefits respondents perceived of the respective energy technologies (*β*_NP_ = 0.64, *p* < 0.001; *β*_SG_ = 0.56, *p* < 0.001; Hypothesis 1b), the more they evaluated NP and SG as an acceptable technology. In line with Hypothesis 2, perceived benefits of both NP (95% CI: 0.68–0.60) and SG (95% CI: 0.80–0.52) more strongly contributed to explaining the acceptability of the respective energy technology than perceived risks (95% CI_NP_: –0.3––0.25; 95% CI_SG_: –0.4––0.35), as shown by the 95% CIs that did not overlap at all.

Together with benefit perceptions, social network characteristics explained 61% of risk perceptions toward NP (*p* < 0.001; *ƒ* ^2^ = 1.78), and 63% of risk perceptions toward SG (*p* < 0.001; *ƒ* ^2^ = 1.70), both representing a strong effect (Hypothesis 3). Results showed that the more respondents perceived support toward the energy technology in a person's social network, the less risky they perceived the technology (NP: *β* = –0.14, *p* < 0.001; SG: *β* = –0.18, *p* < 0.001), hereby supporting Hypothesis 3a. In line with Hypothesis 3b, the more people a person talked about risky energy technologies in their network, the more risks they perceived toward NP (*β* = 0.05, *p* < 0.01) and SG (*β* = 0.08, *p* < 0.001).

Social influence also contributed to the explanation of benefit perceptions of NP (*R* ^2^ = 0.25, *p* < 0.001; *ƒ* ^2^ = 0.33) and SG (*R* ^2^ = 0.23, *p* < 0.001; *ƒ* ^2^ = 0.30), representing a strong effect. The more people perceived support of NP (*β* = 0.50, *p* < 0.001) or SG (*β* = 0.48, *p* < 0.001) in their social network, the more benefits they assigned to NP and SG, hereby supporting Hypothesis 3c. Talking about risky energy technologies in one's social network did not positively affect benefit perceptions of NP (*β* = –0.00, n.s.) and SG (*β* = –0.04, n.s.), hereby rejecting Hypothesis 3d.

With regard to Hypothesis 4, we tested the indirect effects of the social network factors on the acceptability of the two energy technologies with risk and benefit perceptions as mediator constructs. Table II presents the estimates for the PLS path model for both technologies. The mediator effect of risk perceptions between perceived support in one's social network toward the energy technology and the acceptability of the technology was supported (NP: *β* = 0.04, *p* < 0.001, 95% CI: 0.03–0.06; SG: *β* = 0.07, *p* < 0.001; 95% CI: 0.05–0.10). The mediator effect of risk perceptions between the extent to which people talk about risky energy technologies in one's social network and acceptability was supported as well (NP: *β* = 0.02, *p* < 0.05, 95% CI: –.05–0.01; SG: *β* = –0.03, *p* < 0.01; 95% CI: –0.05–0.01). Hence, risk perceptions mediated the two social network factors for both technologies, hereby supporting Hypothesis 4a.

**Table II:** Mediating Effect of Risk and Benefit Perceptions of Nuclear Power (NP) and Shale Gas (SG): Indirect Effects of Social Influence on Acceptability

	Support→RP→ AC		Talk→RP→AC		Support→BP→AC		Talk→BP→AC	
	NP	SG	NP	SG	NP	SG	NP	SG
Indirect effect (*SE*)	0.04 (0.01)	0.07 (0.01)	−0.02 (0.01)	−0.03 (0.01)	0.32 (0.03)	0.27 (0.02)	−0.00 (0.02)	−0.02 (0.02)
*p*‐Value	<0.001	<0.001	0.02	0.001	<0.001	<0.001	0.88	0.26
95% CI	(0.03–0.06)	(0.05–0.10)	(−0.03–0.00)	(−0.05–0.01)	(0.27–0.37)	(0.22–0.31)	(−0.04–0.03)	(−0.06–0.02)

Support = Perceived support toward nuclear power/shale gas in one's network; Talk = talking about risky technologies in one's social network; RP = risk perception; BP = benefit perception; AC = acceptability of nuclear power/shale gas; NP = nuclear power; SG = shale gas.

The relationship between perceived support in one's social network toward the energy technologies and their acceptability was mediated by benefit perceptions (NP: *β* = 0.32, *p* < 0.001, 95% CI: 0.27–0.37; SG: *β* = 0.27; *p* < 0.001; 95% CI: 0.22–0.31). However, the relationship between the extent to which people talk about risky energy technologies in one's social network and acceptability was not significantly mediated by benefit perceptions (NP: *β* = –0.00, *p* = n.s., 95% CI: –0.04–0.03; SG: *β* = –0.02, n.s.; 95% CI: –0.06–0.02). Therefore, these results provided partial support for Hypothesis 4b.

### 3.2 Difference in Familiarity: Comparing Models of NP Versus SG

To test Hypothesis 5 and *RQ1*, we conducted a Multigroup Analysis via a bootstrap procedure. Table III shows that most path coefficients were not significantly different from one another, with two exceptions. Our findings showed that risk perceptions affect the acceptability of the more familiar risk technology of NP (*β* = –0.30) less strongly than of the less familiar technology of SG (*β* = –0.39; *t*(947) = 2.11, *p* < 0.05), hereby supporting Hypothesis 5a. Furthermore, benefit perceptions more strongly affected the acceptability of NP (*β* = 0.64) than SG (*β* = 0.56; *t*(947) = 1.89, *p* = 0.059), hereby supporting Hypothesis 5b. Social influence factors did not affect risk and benefit perceptions differently for more (NP) or less (SG) familiar technologies, hereby answering *RQ1*.

**Table III:** Differences of Path Coefficients for a Familiar (Nuclear Power) and an Unfamiliar (Shale Gas) Risky Energy Technology

Structural Effect	Nuclear Power	Shale Gas	Difference	*t* (*p*‐Value)
RP →Acceptability	−0.30	−0.39	0.10	2.11 (0.035)
BP →Acceptability	0.64	0.56	0.08	1.89 (0.059)
Support → RP	−0.14	−0.18	0.04	1.18 (0.240)
Talk to → RP	0.05	0.08	0.02	0.73 (0.463)
Support → BP	0.50	0.48	0.02	0.51 (0.611)
Talk to → BP	−0.00	−0.04	0.03	0.73 (0.465)

Support = Perceived support toward energy technology in one's network; Talk = talking about risky technologies in one's social network; RP = risk perception; BP = benefit perception.

## 4 DISCUSSION AND CONCLUSION

Risky energy technologies are often controversial and debates around them are polarized. In debates surrounding risky energy technologies, public acceptability is a key issue. Previous research shows that risk and benefit perceptions influence public acceptability of energy technologies (Visschers et al., 2011). Within this research, there has been a strong emphasis on intrapersonal factors, such as values (De Groot et al., 2013) and knowledge (Helgeson et al., 2012) influencing these relationships. This research integrates interpersonal factors (i.e., social influence measured through social network characteristics) with two energy technologies that differ in familiarity, NP versus SG, to examine how these factors explain risk and benefit perceptions and public acceptability. By integrating interpersonal with intrapersonal factors in an explanatory model, we show how social influence explains acceptability judgments and how they could extend the current “risk–benefit acceptability” model used in the field of risk research.

In line with Hypothesis 1, our findings show that higher risk perceptions of NP and SG lead to a decrease in the acceptability of the respective energy technology. Inversely, higher benefit perceptions are related to an increase in the acceptability of NP and SG. This finding supports previous studies related to the acceptability of risky energy technologies (e.g., Bearth et al., 2014, 2017; Siegrist et al., 2008). The findings extend current literature in replicating this relationship for the lesser familiar energy technology of SG.

The perceived benefits of NP and SG are more important in informing acceptability judgments than the perceived risks associated with them, hereby supporting Hypothesis 2. Previous studies have pointed to the superior role benefit perceptions play over risk perceptions in people's acceptability of hazards (Siegrist et al., 2008). These findings seem to be robust, as they hold up regardless of participants` familiarity with the specific energy technology. Gaskell et al. (2004) have argued that the strong reliance on benefit perceptions can be attributed to the lexicographic heuristic in which people base their attitude on the single most important attribute of the attitude object. The benefits of NP and SG alike are that both have been argued to be cheap and independent energy technologies for households and the economy (BBC, 2013; Boudet et al., 2014). These benefits may have a more direct influence on the individual than the risks associated with NP and SG, which could be detrimental to the environmental and human health on a short and long‐term basis (Howarth, Ingraffea, & Engelder, 2011; Siegrist & Cvetkovich, 2000). Thus, the formation of acceptability may be most strongly influenced by benefit perceptions because the benefits of these energy technologies impact people's lives more directly than do the risks associated with these energy technologies.

Our findings support the assumption that social influence affects risk and benefit perceptions of NP and SG (Hypothesis 3). The present study operationalized social influence in two ways. First, it is operationalized as the percentage of the total number of close network peers that were perceived to support NP and SG. Second, the study assessed the amount of people an individual talk to about risky energy technologies such as NP and SG in one's close network. Our results show that the more individuals perceive support in their social network toward NP or SG, and, the more they talk about energy technologies in their network, the fewer risks they perceive toward the technology. Similarly, the more individuals perceive support in their social network toward NP or SG, the more benefits they perceive toward the technology. However, talking about energy technologies in one's social network did not explain benefit perceptions for NP or SG. Overall, the results support Scherer and Cho's (2003) social network contagion theory of risks. This theory argues that relational aspects of an individual's network influence and form an individual's perception. The strong relationships between social influence and risk and benefit perceptions are also coherent with previous findings showing that risk perceptions of individuals are heightened when their social network peers display concern about the attitude object (Kohler et al., 2007).

Our findings show that perceived support in one's social network toward the risky energy technology was especially relevant for explaining risk, and, even more so, for benefit perceptions. Hence, social network peers that support an energy technology are most likely to pass on their belief system to their network members. This is coherent with the finding that benefit perceptions are the strongest predictor of acceptability Finucane et al., 2000; Siegrist & Cvetkovich, 2000). Furthermore, these results suggest that attitudes are most strongly affected by knowing and sharing actual beliefs about the positive, and, to a lesser extent, negative views of the energy technology. This occurs through the exchange of opinions and sharing perceptions regarding the energy technologies, rather than only talking about risky energy technologies in general. This finding emphasizes that it is more the beliefs than the exchange in information itself between peers that influence one's own attitude.

Previous research suggests that most determinants of the acceptability of risky energy technologies exert their influence via risk and benefit perceptions (Siegrist & Cvetkovich, 2000). This is indeed the case for intrapersonal factors, such as values (De Groot et al., 2013) and knowledge (Helgeson et al., 2012). Our findings show that this process works similarly for interpersonal factors: we found that social influence, as measured by social network characteristics, affects public acceptability of energy technologies indirectly via risk and benefit perceptions (Hypothesis 4). These results contribute by demonstrating the extent to which, and under which circumstances, social influence explains public acceptability of highly controversial and debated energy technologies, which are different in familiarity. The findings provide a starting point to further develop the previous well‐established “risk–benefit acceptability” model as proposed in risk research (e.g., Ho et al., 2018; Lienert et al., 2015; Visschers et al., 2011) by investigating its underlying processes.

Overall, the study demonstrates that risk and benefit perceptions are differently related to the acceptability of the familiar risky energy technology of NP compared to the unfamiliar technology of SG (Hypothesis 5). Risk perceptions are relatively more important for explaining the acceptability of SG than NP, while benefit perceptions more strongly affect the acceptability of NP than SG. These results are in line with Van Giesen et al. (2018), which suggests that when people are relatively unfamiliar with the risky technology, they tend to rely more on biases, such as the negativity bias (Baumeister et al., 2001), as there are less knowledge structures in place to rationalize negative information. However, over time, when knowledge structures are in place, the negativity bias might play less of a role. Therefore, future studies could take a longitudinal approach to examine whether the differences in the relative contribution of risk and benefit perceptions in NP and SG will diminish over time, as the knowledge structures, and, therefore, the perceived familiarity for SG grows.

Finally, our findings do not provide evidence that social influence works significantly different depending on the familiarity with the context (*RQ1*). It therefore rejects the idea that people's attitudes are more easily influenced by others when they are less familiar or more uncertain with an attitude object (Christenson et al., 2017). These results support the robustness of our conceptual model as the familiarity with the energy technologies does not seem to change the importance of social influence factors, as measured with social network characteristics, on risk and benefit perceptions and acceptability. Therefore, it seems that including social dimensions by integrating interpersonal factors (e.g., social network characteristics) in more established risk–benefit acceptability models is a fruitful way to understand the process of how the acceptability of risky attitude objects is established.

### 4.1 Limitations, Future Research, and Implications

Our results show that including a broader social context in the form of social network characteristics, rather than focusing on the social context from an intrapersonal perspective (e.g., social norms), can be a fruitful way to understand the formation of beliefs, attitudes, and behavior. However, two potential limitations should be addressed in future studies. First, the exploratory and correlational nature of the present study prevents us from understanding through which mechanisms social networks influence these beliefs, attitudes, and behavior. Thus, limiting the conclusions we can draw from our findings. Future research could take our findings as a point of departure to examine these mechanisms in more in‐depth. One way forward would be to integrate important intrapersonal (social) factors with (interpersonal) social network characteristics to examine their interactions. For example, certain social network characteristics might “trigger” social norms in favor (perception that most people in your close network are supporting the technology) or against (perception that most people in your close network are opposing the technology) risky energy technologies, hereby strengthening the relationship between social norms, beliefs, and behavior (see, e.g., the Theory of Normative Conduct; Cialdini et al., 1990). In line with this, we would urge future research to further examine the causal direction under which opposition and support in one's social network is most relevant by taking a more experimental or longitudinal approach.

Second, in the present study, we argue that social network factors could be regarded as interpersonal factors, while social norms have typically been considered to be an intrapersonal factor in past research. Although conceptually social network factors are on an interpersonal level, our present study has measured social networks on an egocentric level, which, like social norms, is based on the individual's perception of the views of their peers. Still, there is an essential difference in the operationalization between social networks and social norms. Social norms are most often measured as the summation of normative beliefs from several salient others (cf. Ajzen, 1985). However, research in social norms has found that the nature of the social norm depends on whom the norm is derived from (Keizer & Schultz, 2019). That is, the nature of the influence from others depends on who those relevant others are, which are often not further investigated in research (see, e.g., Farrow, Grolleau, & Ibanez, 2017). Using egocentric social networks can be regarded as a way to further conceptualize and operationalize these “relevant others.” Specifically, in the egocentric approach we applied, individuals are asked to report the perception of NP and SGfor each one of their close network members. Hence, researchers get a more detailed picture following this approach than when they assess the social norm as an entire network. From a more practical point of view, it has been suggested that complying with norms is most likely to emerge when people interact in small homogeneous communities or in networks that can create these conditions (Kinzig et al., 2013). When research only focuses on measuring social norms rather than the detailed networks, interventions based on social norms are less likely to succeed. Thus, although the present social network characteristics are conceptualized on an interpersonal level but operationalized on an intrapersonal level, it includes detail and systematic analysis about an individual's network. Hence, it may help our understanding of conceptualizing and operationalizing social influence. However, to further align the conceptualization and operationalization of social network factors, future studies should include alter social network analyses as well.

Individuals’ benefit perceptions have a larger impact on the acceptability of risky energy technologies, such as NP and SG, than risk perceptions. This influence is particularly pronounced for forming ones’ acsceptability of more familiar energy technologies. Communication strategies of businesses and policy makers, wishing to increase the acceptability of relatively familiar technologies, should therefore focus on emphasizing their benefits. However, for less familiar risky technologies, our findings suggest that emphasizing the perceived risks of (not) implementing the energy technology will influence its acceptability as well. Addressing perceived risks may also further reduce the danger of public polarization of opinions. The strong correlation between risk and benefit perceptions as found in our study suggests that reducing perceived risks may also heighten benefit perceptions, which in turn strengthens public acceptability as well. Consequently, addressing risk and benefit perceptions likely affects how individuals perceive the support toward risky technologies in their social network and how the narrative will be shaped in their social influence context (i.e., how they perceive their network members to think about these technologies). Thus, addressing risk and benefits at an individual level will not only change the opinion of a single person, but instead may have social influence effects on others that form their attitudes based on opinions present within their social network.

Public acceptability of risky energy technologies is multifaceted and depends on different inter‐ and intrapersonal factors. Present research shows that social influence plays an essential role in evaluating risks and benefits of NP and SG, and indirectly, public acceptability. However, risk perceptions are more important for unfamiliar energy technologies such as SG, while benefit perceptions are more important when explaining the acceptability of more familiar risky energy technologies. Our findings help future research to develop more comprehensive models of acceptability formation of energy technologies through the integration of social influence and different energy technologies, hereby contributing to the fields of risk research and social influence.

## Supporting information


**Table SI**. Review of the Revised Measures.
**Table SII**. Standardized Path Coefficients for Old and New Acceptability Construct with Perceptions of Risk and Benefits (*N* = 153).
