# Supplementary material for: Social Influence, Risk and Benefit Perceptions, and the Acceptability of Risky Energy Technologies: An Explanatory Model of Nuclear Power Versus Shale Gas
Source: Risk Anal. 2020 Feb 13;40(6):1226–43. doi: 10.1111/risa.13457 (PMC7317191; doi:10.1111/risa.13457)
Supplement: Supplementary file 1 — Table SI. Review of the Revised Measures. Table SII. Standardized Path Coefficients for Old and New Acceptability Construct with Perceptions of Risk and Benefits (N = 153). [file RISA-40-1226-s001.docx]

**Supplementary file**

**Background:** The reviewers voiced two issues regarding the measurement of our psychological constructs in our main study as reported in the present paper. Specifically, they were concerned about the dependent variable “acceptability”. The scale included two items that used the word “reluctantly” (see Table 1) which could be perceived as ambivalent. They also pointed out the fact that the scale items of the independent variable “familiarity”, measuring the difference between the familiarity of nuclear power versus shale gas, did not entirely match the response scale. Therefore, we decided to collect some more data, with some new items and a changed the familiarity scale to evaluate the validity of our findings.

**Objective:** Thus, the objective of the additional data collection was to examine to what extent the concerns with our measures of acceptability and familiarity might have affected our main study’s findings (as reported in the paper).

**Method:** To address the concerns in relation to the measures used in the study reported in the paper, we conducted a second small-scale survey (*N*=153, with MTurk; sampling method and sample characteristics similar to the main study), including three alternative acceptability items adopted from Tsujikawa et al. (2016) in addition to our initial acceptability items. We also revised the two acceptability items by removing the word “reluctantly”. Additionally, we revised the familiarity construct by matching the response scale to the items. We also included an extra familiarity item (“How knowledgeable are you with the risks and benefits of NP/SG?” (scale ranging from ‘1- not knowledgeable at all’ to ‘5- very knowledgeable’). Our reasoning behind this was to add a knowledge-dimension to measure familiarity, as we had implied a conceptual overlap between the two throughout our literature review. See Table 1 for a review of the revised measures.

Table 1. Review of the Revised Measures

| ***variable*** | ***Items*** | ***Source*** |
| --- | --- | --- |
| Acceptability “old” | 1. The UK needs a lot of electricity; people should therefore accept NP.  2. I *reluctantly* accept that we will need NP to help combat climate change.  3. I am in favor of NP to be part of the of the UK’s energy mix in 2025.  4. I *reluctantly* accept that we will need NP to help improve energy security in the UK. | Visschers et al. (2011)  Corner et al. (2011)  O’Hara et al. (2014)  Corner et al. (2011) |
| Acceptability “new” | 5. I am in favour of NP for energy generation  6. NP is an excellent way to generate electricity  7. The number of NP plants in the UK should be increased | Tsujikawa et al. (2016)  Tsujikawa et al. (2016)  Tsujikawa et al. (2016) |
| Familiarity | 1. How familiar are you with the risks and benefits of NP?  2. How much have you ever heard or read about NP?  3. How knowledgeable are you about the risks and benefits of NP? | Boudet et al. (2014)  Boudet et al. (2014)  Extra item based on our conceptualization of familiarity |

*Notes.* The items are shown for nuclear power (NP). The questions assessing shale gas used the same wording only replacing “nuclear power” with “shale gas”. Acceptability items were measured on a Likert scale ranging from 1 ‘strongly agree’ to 5 ‘strongly disagree.’ The words in *Italics* were included in the study as reported in the paper, but deleted in the final measurement of the new study as it expressed ambivalence. Familiarity items were measured on 5-point semantic differential scales (‘not familiar at all’-‘extremely familiar’; ‘very little’ – ‘very much’; ‘not knowledgeable at all’ – ‘very knowledgeable’).

**Results:** The main results regarding the measures in concern include:

1. The old and the new acceptability measures were both reliable (Cronbach’s α_old_ = .89 versus Cronbach’s α_new_ = .88) and were also strongly correlated with one another (*r* =.84), implying they all measured the same underlying theoretical construct.
2. The construct of familiarity showed similar reliabilities as in the main study. Also, the extra item of familiarity was strongly related to the initial two items (*r* = .77 and *r* = .73). The three items formed a reliable construct (Cronbach’s α = .85). Furthermore, the difference in familiarity between NP and SG with the revised measure was in the expected direction and in line with our main study results (i.e., NP: *M*=3.5, *SD*=0.9 versus SG: *M*=2.9, *SD*=1.1; *t*(304)=5.1, *p*<.001). Thus, the results replicate the findings of the main study reported in the paper and therefore the validity of the measurement scale as used in our main study.
3. Naturally, we had some slight variations in results. However, in line with our observations above, we can report that the main findings of the measurement and structural model did not change dramatically between the two studies, when using the old or the new measures (see Table 2). Indeed, the results of the new study were very similar so that we did not have to change our conclusions in relation to our conceptual model based on the new results.

Table 2. Standardized path coefficients for old and new acceptability construct with perceptions of risk and benefits (*N*=153).

|  | Nuclear power | | Shale gas | |
| --- | --- | --- | --- | --- |
| Acceptability | *old* | *new* | *old* | *new* |
| Risk Perception | -.30 | -.11 | -.43 | -.40 |
| Benefit Perception | .62 | .72 | .53 | .50 |
| *R*^2^ | .72 | .64 | .82 | .72 |

**Conclusion:** In conclusion, our additional data collection with amended items makes us feel confident that our operationalizations of constructs have not influenced the main conclusions of our reported findings.

**Reference**

Tsujikawa, N., Tsuchida, S., & Shiotani, T. (2016). Changes in the factors influencing public acceptance of nuclear power generation in Japan since the 2011 Fukushima Daiichi nuclear disaster. *Risk Analysis, 36*(1), 98-113.
